# Completely predatory development is described in a braconid wasp

**DOI:** 10.1038/s41598-022-05705-x

**Published:** 2022-02-02

**Authors:** A. P. Ranjith, Donald L. J. Quicke, K. Manjusha, Buntika A. Butcher, M. Nasser

**Affiliations:** 1 Insect Ecology and Ethology Laboratory, Department of Zoology, University of Calicut, Kerala, 673635 India; 2 Integrative Ecology Laboratory, Department of Biology, Faculty of Science, Chulalongkorn University, Phayathai Road, Pathumwan, Bangkok, 10330 Thailand; 3 Center of Excellence in Entomology: Bee Biology, Diversity of Insects and Mites, Chulalongkorn University, Phayathai Road, Pathumwan, Bangkok, 10330 Thailand; 4 Systematic Entomology Laboratory, Malabar Christian College, Kozhikode, Affiliated to University of Calicut, Kerala, 673001 India; 5 Present Address: Insect Biosystematics and Conservation Laboratory, Ashoka Trust for Research in Ecology and the Environment (ATREE), Royal Enclave, Srirampura, Jakkur Post, Bangalore, 560064 India

**Keywords:** Zoology, Ecology

## Abstract

Hymenopteran parasitoids are well known for their ubiquitous diversity, important ecological roles and biocontrol potential. We report the first detailed documentation of mite predation by a parasitoid wasp, *Bracon predatorius* Ranjith & Quicke sp. nov., (Insecta: Hymenoptera), first case of obligate predatory behaviour in the family Braconidae and first case of mite feeding within the superfamily Ichneumonoidea. Larvae of a new wasp species are shown to develop entirely as predators of eriophyid mites that induce leaf galls in a commercially important plant. They display highly modified head capsule morphology that we interpret as being associated with this atypical life style. We propose that the new feeding strategy evolved separately from recently described entomophytophagy in another species of the same genus. The divergent larval morphological adaptations of both species indicate a high degree of evolutionary developmental plasticity in the developmental stage.

## Introduction

The enormously diversified order Hymenoptera includes sawflies, bees, wasps and ants, and is one of the four largest insect orders^1,2^. The vast majority of hymenopterans are parasitoids of other insects and thus are often used by us humans as biological-management agents^3^. Although the parasitoid life-history strategy has evolved in diverse insect groups, recent estimates show that 10–20% of all known insects are parasitoid wasps^4^. The parasitic Hymenoptera exhibit either of two mode of development, ecto- or endoparasitoidism, the former generally being the plesiomorphic condition^5^. Ectoparasitoids lay their eggs on or close to the host after paralyzing it. Complete host paralysis leads to idiobiosis^6^ in which the parasitoid larva’s interaction with the host is short lived. Most idiobiont parasitoids attack hosts that are concealed^4^. Species of the highly diverse superfamily Ichneumonoidea (comprising Braconidae, Ichneumonidae and Trachypetidae) are predominantly parasitoids on other insects^7^, although a few display other biologies including gall induction and seed predation^8,9^, entomophytophagy^10^ or predation within arachnid egg masses^11^. Most species of Braconidae are parasitoids of larval Lepidoptera, Diptera, and Coleoptera^12^. Egg predation has evolved on several occasions within the Ichneumonidae where it is clearly derived from the parasitoid ground plan, but predation of multiple mobile prey is only known from one cryptine species^13^.

**Figure 1:**
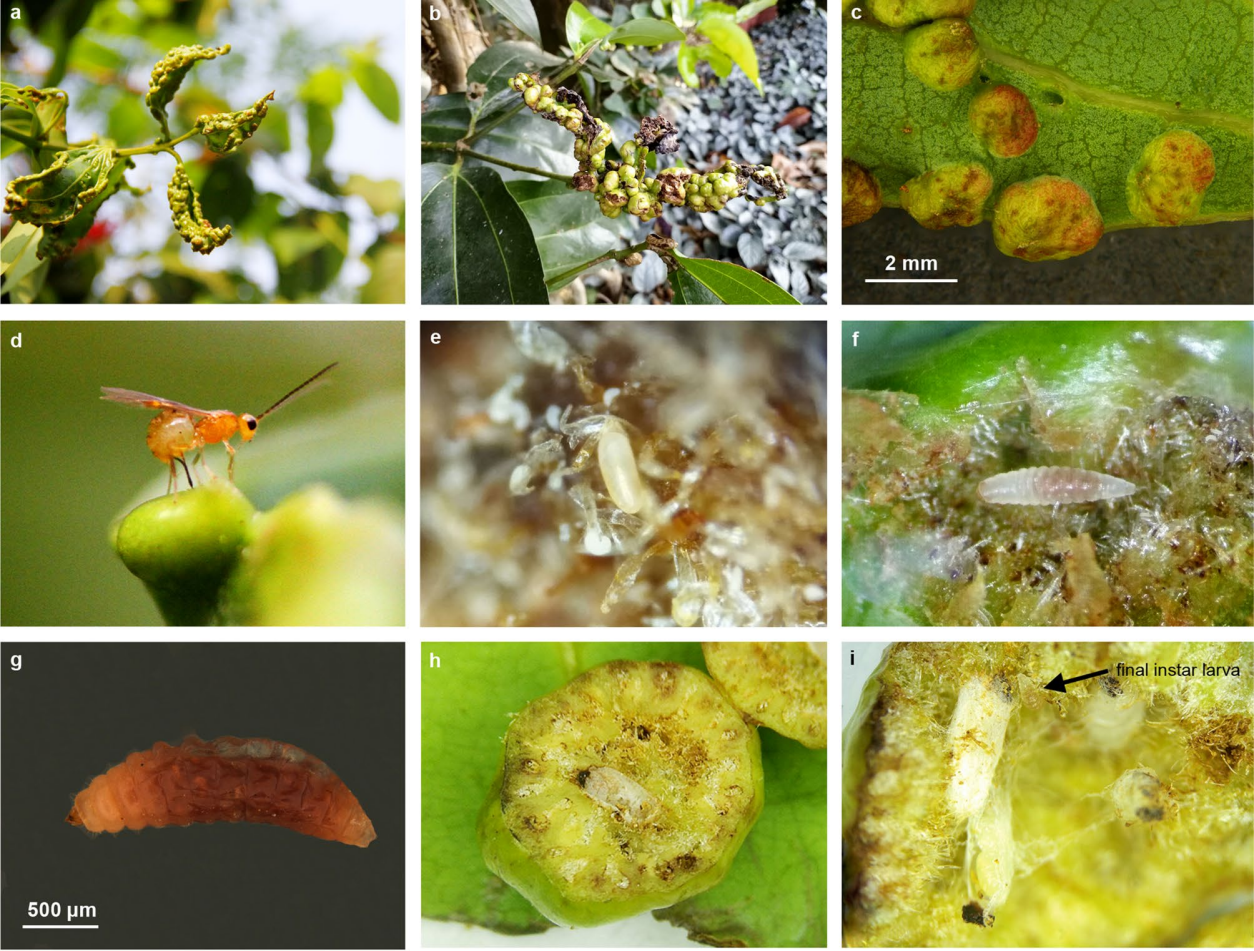
Leaf galls induced by *Aceria doctersi* (Nalepa) on *Cinnamomum verum* J.Presl. and life history of *Bracon predatorius* Ranjith & Quicke sp. nov. **(a–c**) Leaf galls of *C. verum* induced by *A. doctersi* (**a**) young leaf galls, (**b**) mature and dehisced leaf galls, (**c**) light microscopic image of leaf galls, (**d–i**) biology of *B. predatorius* (**d**) adult female wasp ovipositing into the leaf gall, (**e**) egg, (**f**) final instar larva, (**g**) light microscopic image of final instar larva, (**h**) cut opened gall with single pupa, (**i**) larva and pupae in situ.

We provide here the first detailed observations of acarophagy, a new larval feeding strategy, in the parasitoid Hymenoptera, the first such case in the Ichneumonoidea, and the first case of purely predatory behaviour in the Braconidae. In addition to this, we describe a new braconid wasp, *Bracon predatorius* Ranjith & Quicke sp. nov. (Insecta: Hymenoptera). The larvae of this wasp feed solely on the mite, *Aceria* (=*Eriophyes*) *doctersi* (Nalepa, 1909) (Acari: Eriophyidae) which induce galls on the leaves of *Cinnamomum verum* J.Presl (Lauraceae)^14^ (Fig 1a–c) an economic crop in much of S.E. Asia^15^. Severe infestation by the mite leads to a high density of galls which reduces plant vigour (Fig 1a,b). The biology described here expands both host range and feeding strategy within the Ichneumonoidea.

## Results

The presents study indicates that *Bracon predatorius* generally oviposits during early stages of gall development (Fig. 1d) on galls induced by *Aceria doctersi* mostly on tender leaves (Fig 1a–c) and rarely on petioles and stems^13^. The number of *B. predatorius* larvae in parasitized galls ranged from 1–27 (n=93). Eighty-five percent of the examined galls (n=109) were parasitized by *B. predatorius*. Different development stages of larvae (Fig. 1f,g) and pupae (Fig. 1i) of *B. predatorius* were found together in some large galls (n=31) (Fig. 1i), which suggests multiple oviposition at different stages of gall development. Dissection of leaf galls two hours after oviposition by *B. predatorius* always revealed only a single egg (n=8). No live *A. doctersi* individuals were found close to the parasitoid wasp pupae (Fig. 1h). *Aceria doctersi* galls parasitised by *B. predatorius* have also been found in Kodakara (Thrissur district, Kerala) about 100 km away from the type locality in Kozhikode.

**Figure 2:**
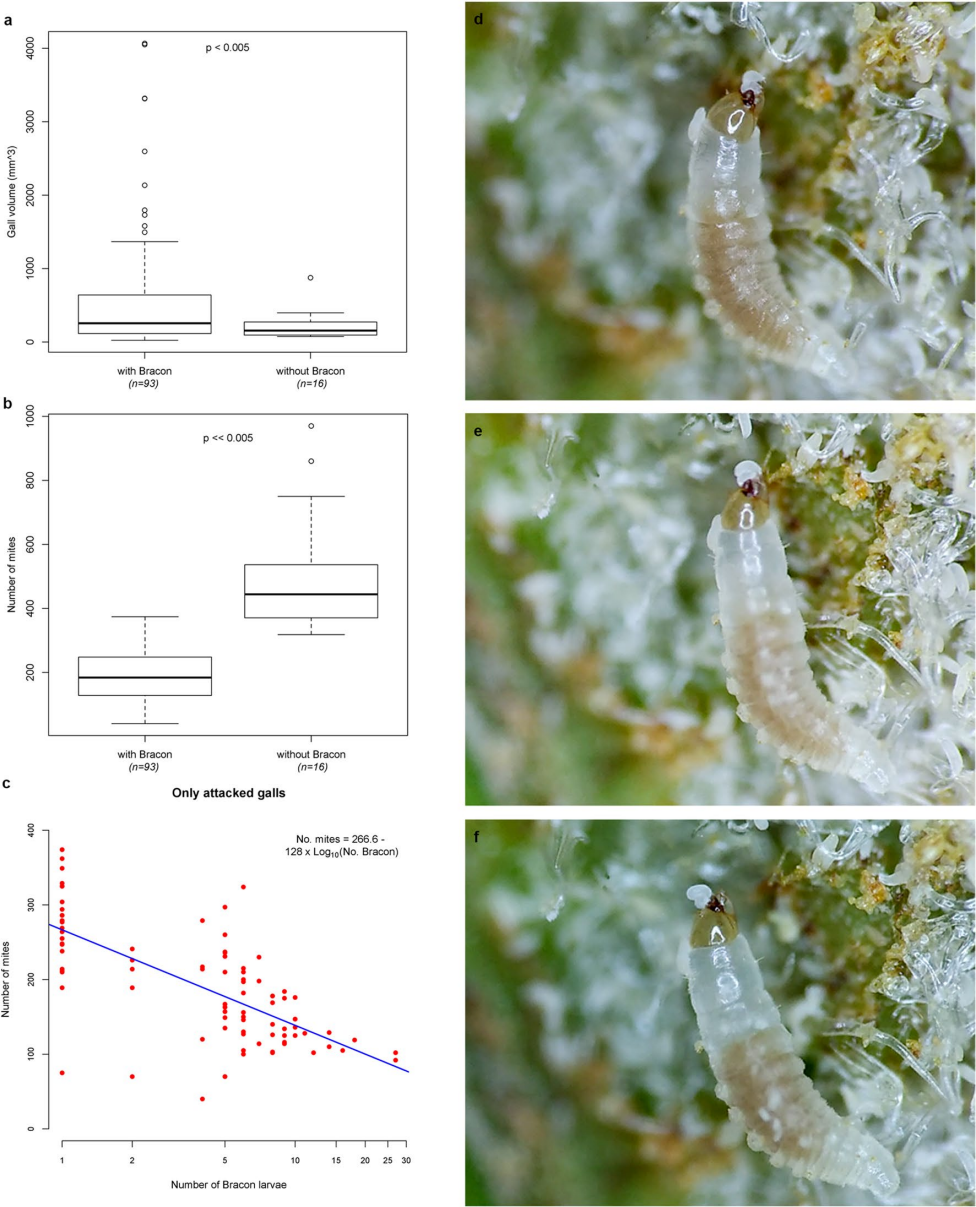
Predatory behaviour of *Bracon predatorius* Ranjith & Quicke sp. nov.** (a–c**) Relationships between presence/absence and number of *B. predatorius*, gall size and numbers of mites (median, upper and lower quartiles, 1.5 × interquartile range and outliers): (**a**) galls without *Bracon predatorius* (n = 16) are significantly smaller than those with one or more *Bracon predatorius* (n = 93) (t = 3.7592, DF = 97.265, p-value = 0.000291), (**b**) galls without *Bracon predatorius* contain significantly more mites than those with (t = 6.308, DF = 15.877, p-value = 0.0001), (**c**) mite number as a function of number of *Bracon predatorius* larvae (only in parasitised galls) with gall volume as co-variate (n = 93, adjusted R^2^ = 0.4657,F = 21.13 on 3 and 89DF, p-value = 0.0001), gall volume and interaction were non-significant. (**d–f**) Sequential images of predatory behaviour of *Bracon predatorius*.

**Figure 3:**
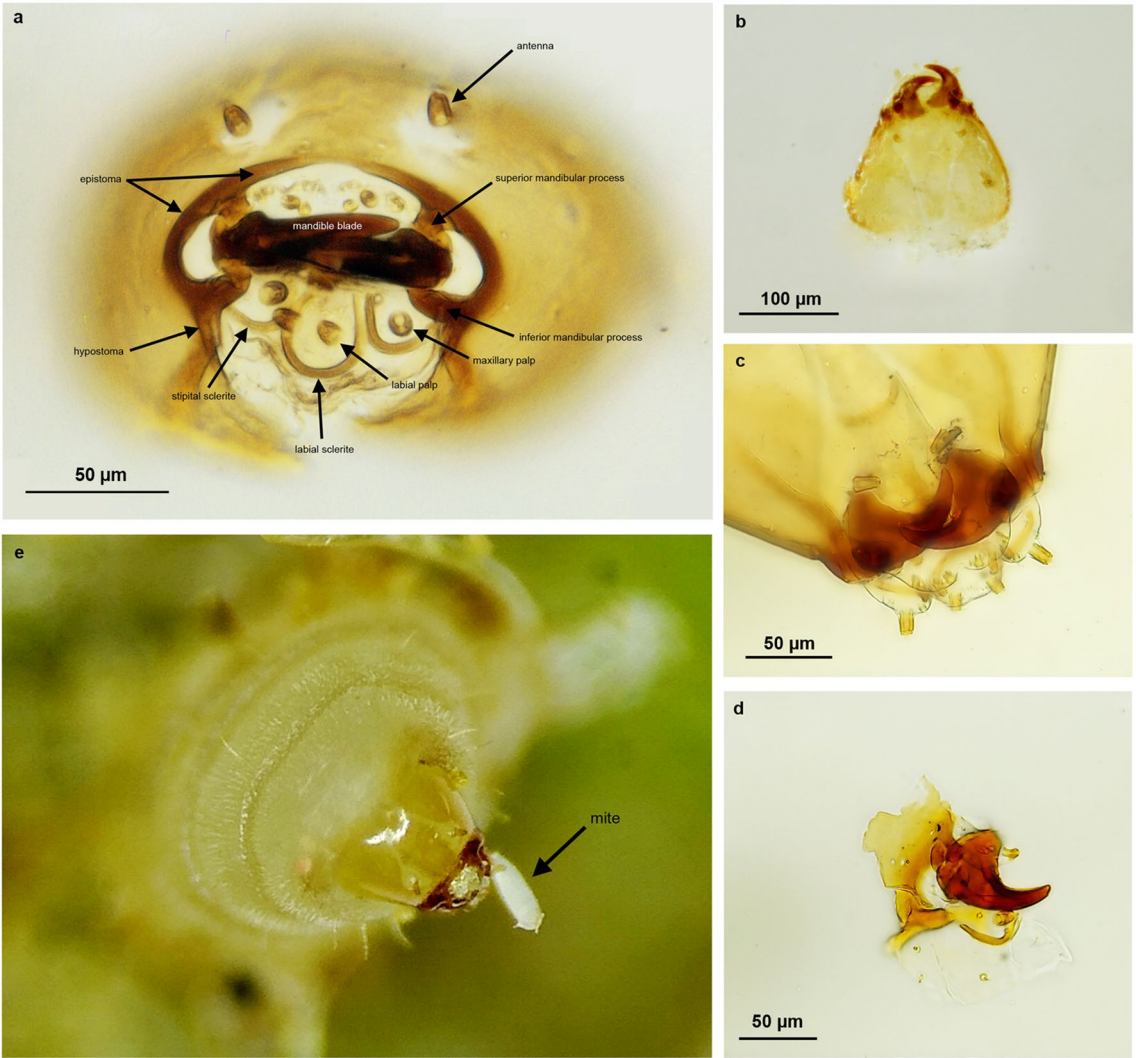
Final instar larval cephalic structure of *Bracon predatorius* Ranjith & Quicke sp. nov. **(a–d**) Slide microphotographs of larval head capsule and mandible (**a**) macerated head capsule in anterior view, (**b**) head capsule, in dorsal view, (**c**) head capsule (in part), ventral view, (**d**) right mandible, in dorsal view, (**e**) anterior view of living final instar larva of *B. predatorius* consuming mite.

The larval stages of *B. predatorius* feed on both juvenile and adults of *A. doctersi* (Fig 2d–f, Supplementary Video 1) which usually remain close to the erineal hairs on which they feed^16^; no egg predation occurs. Young larvae of *B. predatorius* wriggle through in between erineal hairs (Supplementary Video 1). They use their sickle-shaped mandibles (Fig 3b–e) to hunt mites (Supplementary Video 1). Continuous outward and inward movement of mandibles of *B. predatorius* larvae occurs along with the wriggling movement (Supplementary Video 1). The final instar larvae of *B. predatorius* are the most active and they feed voraciously at the rate of 5–7 *A. doctersi* individuals/min (n=8) (Supplementary Video 1).

Unattacked galls were significantly smaller than those containing *B. predatorius* (means 217 and 595 respectively; p<0.0001) (Fig. 2a) as were galls containing only a single *Bracon* juvenile (p<0.0001). However, galls without *Bracon* larvae contained significantly more mites than either all attacked galls (means 503 and 194 respectively; p<0.0001) (Fig. 2b) or those attacked by only one *Bracon* larvae (p<0.0001). Considering only attacked galls with numbers of *Bracon* and gall volume as explanatory variables and including an interaction term, we found that the number of mites was highly significantly negatively correlated (p<0.0001) with the number of *Bracon* present (Fig. 2c) but gall volume and the interaction terms were both insignificant (p=0.94 and p=0.58 respectively).

*Bracon predatorius* females oviposit singly within leaf galls of size 1.5–2.4 mm during morning (0800–0900 h) and evening hours (1700–1800 h) (Fig. 1d). The searching behaviour of the female *B. predatorius* is completed in 1–3 minutes (n=8). The oviposition is completed in 4–6 minutes (n=8). Adult females of *B. predatorius* oviposit through the adaxial side of the gall-bearing leaves. The egg is deposited in spaces between two adjacent erineal hairs. The *B. predatorius* egg is yellow with rounded edges (Fig. 1e) and is almost as long as the mite*, A. doctersi*. The first instar larva of *B. predatorius* is transluscent and wriggles inside spaces that occur in the galls due to puckering and crinkling of the leaf tissue that includes the erineal hairs. These erinea serve as the source of nutrition for populations of *A. doctersi*. The final instar larva of *B. predatorius* is 1.8–2.1 mm long, dull-white with yellowish head capsule and 13 body segments (Figs 1f,g, 2d–f). The body of the larva of *B. predatorius* bears dorsal protuberances and is densely setose (Figs. 1f,g, 2d–f, 3e). The larvae of *B. predatorius* pupate in the space occupied by the final instar larva (Fig 1h,i), and pupae are oriented in sagittal as well as transverse planes (Fig. 1i). Adults of *B. predatorius* exit by cutting a hole with their mandibles along the dorsal side of the gall.

The wasp larvae possess highly derived cephalic structures: massive, elongate and overlapping mandibles that lack a comb of serrations, and otherwise strongly sclerotised epistoma and well-developed antennae and palps. The cephalic structures of the final instar larva (Fig 3a–e) differ markedly from those of typical ectoparasitic braconines^17^. The epistoma is robust, though weakened medio-dorsally possibly allowing some flexibility, ∩-shaped (Fig. 3a). Both pairs of mandibular processes are large and robust (Fig. 3a). The mandibles are large, heavily sclerotised, with small swollen base and long blades, completely overlapping and without serrated combs (Fig 3a–e). The hypostoma is greatly reduced and the short arms converging ventrally (Fig. 3a). Stipital sclerites are strongly curved, L-shaped (Fig. 3a). The cardo is apparently lacking, or if present, indistinct and weakly sclerotised (Fig. 3d). Antennae, and both maxillary and labial palps are relatively large (Fig. 3a).

Systematic entomology

Hymenoptera Linnaeus, 1758﻿

Apocrita Gerstaecker, 1867

Ichneumonoidea Latreille, 1802

Braconidae Nees 1811

*Bracon* Fabricius 1804

**Figure 4:**
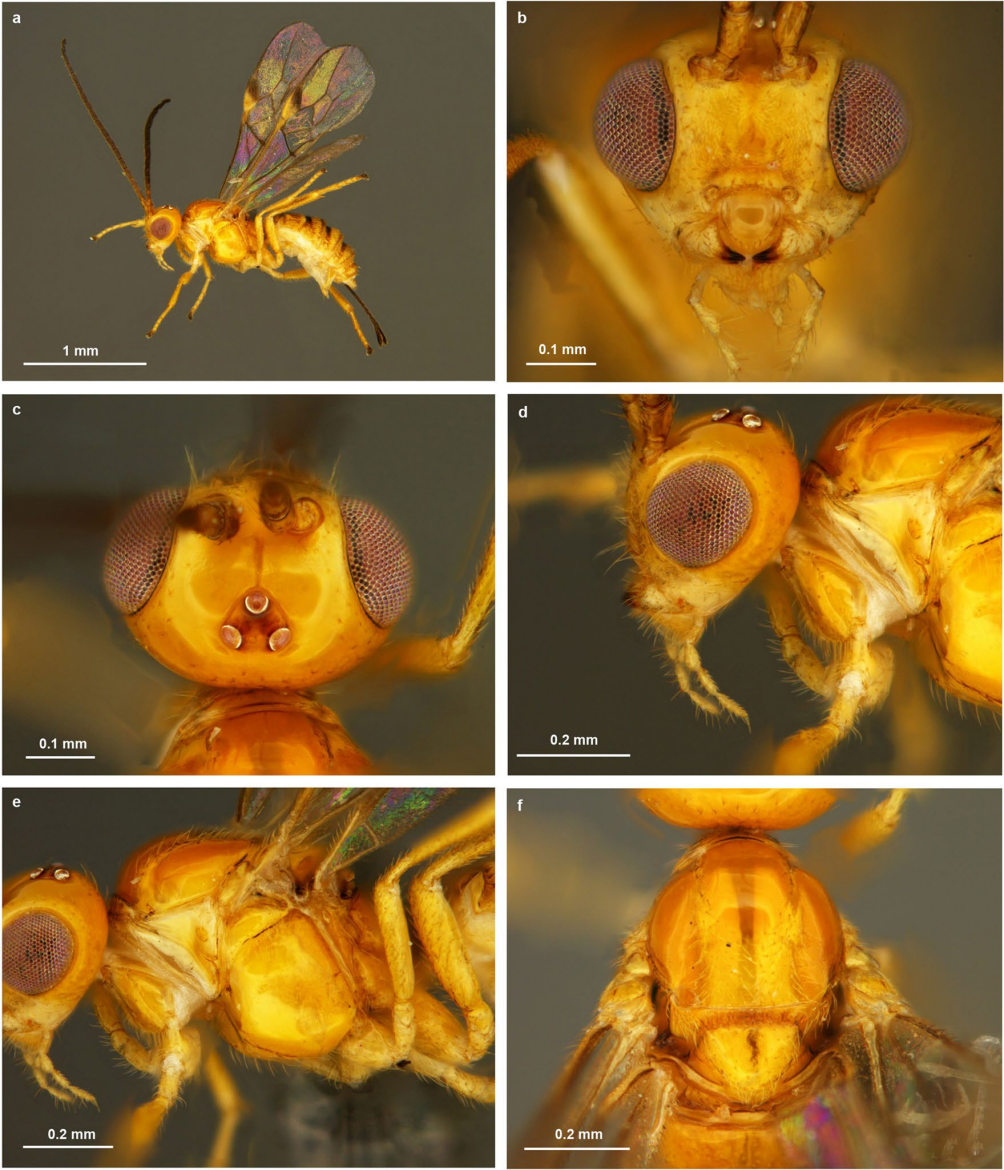
Light microscopic images of *Bracon predatorius* Ranjith & Quicke sp. nov., holotype, female. (**a**) Habitus, in lateral view, (**b**) head, in anterior view, (**c**) head, in dorsal view, (**d**) head, in lateral view, (**e**) mesosoma, in lateral view, (**f**) mesosoma, in dorsal view.

**Figure 5:**
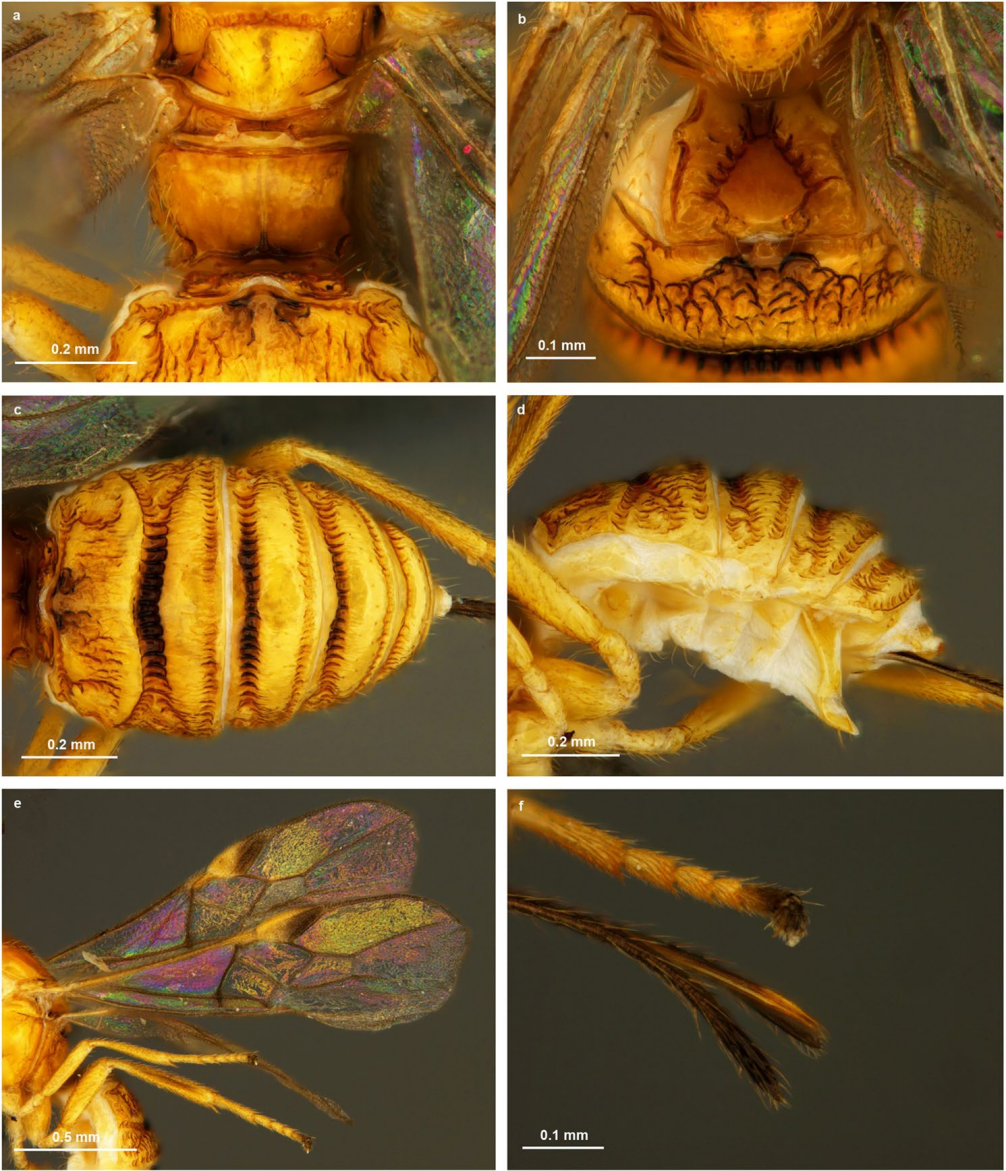
Light microscopic images of *Bracon predatorius* Ranjith & Quicke sp. nov., holotype, female. **(a**) Propodeum, in dorsal view, (**b**) first and second metasomal tergite, in dorsal view, (**c**) metasoma, in dorsal view, (**d**) metasoma, in lateral view, (**e**) wings, (**f**) ovipositor and ovipositor sheath.

*Bracon predatorius* Ranjith & Quicke sp. nov. Figures 4 and 5

### Diagnosis

Body yellow (Fig. 4a). Antenna with 17 flagellomeres. Mandible twisted, two teeth visible in anterior view (Fig. 4b). Face medially smooth, shiny, rest aciculate, sparsely setose (Fig. 4b). Malar groove absent (Fig 4b,d). Frons shiny with distinct midlongitudinal groove (Fig. 4c). Head smooth, evenly rounded posteriorly in dorsal view (Fig. 4c). Median and lateral lobes of mesoscutum largely glabrous, setose posteriorly (Fig. 4f); notauli only indicated anteriorly. Scutellar sulcus narrow, divided by eight carinae. Propodeum smooth with an incomplete midlongitudinal carina, sparsely setose laterally (Fig. 5a). Length of fore wing veins 3RSb: 3RSa: r-rs = 15: 5: 3 (Fig. 5e). Length of fore wing veins 2RS: 3RSa: rs-m = 5: 5: 3. Tarsal claws with pointed basal lobe. Metasoma largely smooth and shiny with seven exposed, sparsely setose, tergites (Fig 5c,d). Median area of first tergite largely smooth and shiny, dorso-lateral carina strong and lamelliform, dorsal carina strong with angulate projection subbasally (Fig. 5b). Second metasomal suture sinuate, strongly crenulate (Fig. 5c); Tergite 4–7 rugose, sparsely setose. Tergite 2–6 with distinct antero-lateral area. Ovipositor slender, darkened apically, with an indistinct dorsal nodus and ventral serrations (Fig. 5f).

### Material examined

Holotype female, “INDIA: Kerala, Malappuram, Calicut University Botanical Garden, 4.iii.2020, emerged from leaf galls of *Cinnamomum verum* induced by *Aceria doctersi*, ex. A.P. Ranjith” (ZSIK-ZSI/WGRC/IR.INV-14852). Paratypes, Two female paratypes and one male paratype with same data as holotype (ZSIK) 20 females and 19 males with same data of holotype (Department of Zoology, University of Calicut (DZUC)).

### Description

Holotype, female.

Length of body 2.1 mm, of fore wing 1.7 mm, and of ovipositor 0.6 mm.

### Head

Antenna with 19 flagellomeres. Terminal flagellomere strongly acute. Median flagellomeres normal in dorsal view. First flagellomere 1.2 times length of second and third flagellomeres respectively, first flagellomere 3.6 times as long as wide. Mandible twisted, two teeth visible in anterior view (Fig. 4b). Inter-tentorial distance: tentorio-ocular distance = 1.5:1. Inter-tentorial distance: height of clypeus = 3:1. Face medially smooth, shiny rest aciculate, sparsely setose (Fig. 4b). Height of eye: shortest distance between eyes: width of head = 1:1.1:2.3. Oculo-antennal groove absent. Malar groove absent. Malar space 0.95 times basal width of mandible. Frons shiny with distinct midlongitudinal groove (Fig. 4c). Stemmaticum triangular forming equilateral triangle. Head evenly rounded posteriorly in dorsal view (Fig. 4c). Length of eye 1.5 times as long as temple in dorsal view. Shortest distance between posterior ocelli: transverse diameter of posterior ocellus: shortest distance between posterior ocellus and eye = 4:4:11.

### Mesosoma

Mesosoma 1.3 times longer than maximum height, largely smooth, shiny (Fig. 4e). Medial lobe and lateral lobes of mesoscutum glabrous, setose posteriorly (Fig. 4f). Pronotum smooth (Fig. 4d). Notauli only indicated anteriorly (Fig. 4f). Scutellar sulcus narrow, divided by eight carinae. Scutellum smooth (Fig. 4f). Median area of metanotum large, smooth, with distinct midlongitudinal and lateral carina anteriorly and stub like longitudinal carina submedially (Fig. 5a). Propodeum smooth with an incomplete midlongitudinal carina, sparsely setose laterally (Fig. 5a).

### Wings

Fore wing, Vein 2-M 1.8 times 3RSa. Vein 1-M straight. Vein (RS+M)a straight (Fig. 5e). Vein rs-m without bulla. Vein 1RS forming an angle of 55° with vein C+SC+R. Vein m-cu 0.6 times 1-M. Vein 1cu-a interstitial. Hind wing vein R1 1.5 times length of 1r-m. Apex of vein C+SC+R with one hamulus. Base of hind wing without medium sized glabrous area distal to vein cu-a on posterior half of cell.

### Legs

Claws with pointed basal lobe. Fore tibia with transverse apical row of thickened bristles. Lengths of hind femur:tibia:basitarsus = 32:46:32.

### Metasoma

Metasoma largely smooth and shiny with six exposed, sparsely setose, tergites (Fig 5b–d). First metasomal tergite 0.86 times as long as wide (Fig. 5b). Second tergite rugose on basal 0.3, rest smooth, 3.1 times wider than medially long, without triangular mid basal area, with a pair of sub lateral depressed area (Fig. 5c). Second metasomal suture sinuate medially, strongly crenulate; third tergite rugose, 4.0 times wider than medially long, without sublateral grooves and with antero-lateral areas defined. Tergite 4–7 rugose, sparsely setose. Tergite 2–6 with distinct antero-lateral area (Fig. 5c). Hypopygium acute apically reaching end of metasomal tergites (Fig. 5d). Ovipositor sheaths 1.2 times longer than hind tibia. Ovipositor slender, darkened sub apically, dorsal valve with an indistinct nodus and serrations ventrally (Fig. 5f).

### Colour

Body yellow (Fig. 4a) except eye grey, stemmaticum silvery, antenna, tip of mandible, anterior 0.5 of pterostigma, wing veins, telotarsus, metasomal suture 2–4, ovipositor sheath light brown to dark brown.

### Male

Similar to female.

### Etymology

The specific epithet refers to its unusual feeding behaviour.

### Remarks

Determination of a new species within the cosmopolitan genus *Bracon* was challenging, indeed there are undoubtedly thousands of undescribed *Bracon* species worldwide. The keys published for the Indian and Chinese species of *Bracon*^18,19^ were unhelpful. The species comes close to *B. keralense* Sheeba & Narendran in having propodeum with midlongitudinal carina not extending to middle of propodeum. Apart from the differences mentioned in the comparative affinities it differs from *B. keralense* in having following characters; length of eye 1.5 times as long as temple in dorsal view (*vs* length of eye 2.30 times as long as temple in *B. keralense*), intertentorial distance 1.5 times tentorioocular distance (*vs* as long as in *B. keralense*), first metasomal tergite with depressed medial area (*vs* with elevated medial area in *B. keralense*). The highly derived larval cephalic structures, which are presumed adaptations to its predatory lifestyle, also differ from the mandibles of all other braconine larvae which have been described to date.

### Comparative affinities

The cosmopolitan genus *Bracon*^7^ includes 17 subgenera and more than 800 described species^20^. For historical reasons, its subgeneric classification is largely based on the Palaearctic fauna and is unreliable especially for extralimital taxa^21–23^. *Bracon predatorius* sp. nov. comes close to the subgenus *Orthobracon* Fahringer although with some distinct differences in the sculpture of propodeum and metasoma, and therefore we provisionally include it under *Orthobracon.* Of the described Indian fauna, *B. predatorius* comes close to *B. keralense* reared from the leaf galls on *Cinnamomum malabatrum* (Burm.f.) J.Presl (Lauraceae) induced by an unnamed Cecidomyiidae^24^, but *B. predatorius* differs in having the face without medial longitudinal ridge, notauli indistinct posteriorly, scutellar sulcus divided by eight carinae, metanotum with midlongitudinal carina anteriorly, second metasomal tergite without smooth, parallel sided medio-basal area, ovipositor without dorsal nodus and ventral serrations.

## Discussion

The present study gives the first detailed documentation of mite predation by a parasitoid wasp *Bracon predatorius* Ranjith & Quicke sp. nov. (Insecta: Hymenoptera), which is the first case of obligate predatory behaviour in the family Braconidae and first case of mite feeding within the superfamily Ichneumonoidea. Even though parasitoid associations of Hymenoptera with non-insect groups is well studied in the past^7,10,25,26^, host–parasitoid associations between the Hymenoptera and Acarina are known only from the superfamily Chalcidoidea^27,28^ and have never been described in detail. Chalcidoid parasitism of non-insect arthropod groups is best known in the family Encyrtidae which includes species of *Ixodiphagus* which are endoparasitoids of ticks^29,30^. Acarophagy within the Hymenoptera has previously only been reported within the chalcidoid family Eulophidae^31,32^. Egg predation of arachnids is also known in a few Ichneumonidae^11,33^, but a rigorous search in the literature did not yield any report of predatory behaviour in the Braconidae nor utilisation of non-insect hosts.

Significant reduction of mites in the galls parasitized by *B. predatorius* confirmed that the obligate acarophagy of *B. predatorius* is enhanced by pure predation of its host mites. This deviating lineage (from parasitoidism) of foraging behaviour (through predation) is likely to be a confirmation of the diversified feeding traits within the 'Parasitica' where aculeates are well known for this feeding trait^34^. Some tentative observations on the predatory behavior by the larvae of the chalcidoid wasp *Aprostocetus* (Eulophidae) within *Eriophyes ribis* (Nalepa) induced plant galls indicate that acarophagy probably also occur within the Chalcidoidea^28,31,32^, however, the larval behaviour of *Aprostocetus eriophyes* has not been described in detail^31^.

In the case of the new Indian predatory *Bracon* species described here, the larval head capsule morphology that we describe is highly derived compared with typical ectoparasitic, idiobiont species of the subfamily^17^. However, highly derived mandibles and other larval features have been described in both the purely phytophagous, seed-predating, *B. phytophagus* and in the entomophytophagous *B*. *garugaphagae*^9,10^. It is suggested that the absence of basal comb of teeth on the mandibles means the larvae feed on the mites without chewing. Most of parasitoid species are either attached onto or located inside their hosts^11^ and some lay their eggs near to the paralysed hosts. It is possible that the pure predatory behaviour reported here for *B. predatorius* is derived from entomophytophagous behaviour as reported in *B. garugaphagae*^10^. Collectively, these observations suggest considerable potential for evolutionary plasticity in braconid larval anatomy as a result of changes in feeding habit.

### Methods collection permits

Necessary permits to conduct sampling of leaf galls from plants (KFDHQ-6561/2019-CWW/WL10) were obtained from the Government of Kerala, India. All experiments with plant and insect were performed in accordance with relevant guidelines and regulations.

### Study site

The study was carried out at two localities: Malappuram (Calicut University Botanical Garden, 11.0800 N, 75.5322 E) and Thrissur (Kodakara district, 10.3838° N, 76.3256 E).

### Sampling and data collection

Samples of *A. doctersi* induced leaf galls on *C. verum* were collected February and March month of 2020. Different developmental stages of leaf galls were dissected in transverse plane under Leica S8 APO stereozoom trinocular microscope. Number of mites and different developmental stages of *B. predatorius* were recorded. Gall volume (mm^3^) was calculated as gall width^2^ × height. Adults and larvae of *B. predatorius* were preserved in 96% ethyl alcohol.

### Species description and terminology

Alcohol preserved adult specimens were treated with hexamethyldisilazane to prevent collapse during drying and then card mounted. Final instar larval head capsules were prepared by macerating the larval heads in 10% KOH (aq. wt/vol.) to dissolve soft tissues, followed by washing in dilute acetic acid, dehydration through alcohol series and mounted in Canada balsam. Morphological terminology used in the description of *B. predatorius* follows van Achterberg^35^ but with wing veins follows Quicke^7^. Terminology for describing sculpture follows Harris^36^. Terminology employed in the description of final instar larval head capsule follows Čapek^17^.

### Illustration and documentation of behaviour

Images from the fields (Fig 1a,b,d) were taken by Canon 7D equipped with 100 mm Canon macro lens. Light microscopic images of egg, larvae, pupa and adults of *B. predatorius* were taken by Leica DMC2900 camera connected with Leica M205A (Figs. 1c,g, 3b, 4 and 5) and Leica DFC295 camera connected with Leica S8 APO (Figs. 1e,f,h,i, 2d–f and 3e) respectively. Images of final instar larval head capsule were taken by Leica DMC4500 camera connected with Leica DM2000 compound microscope (Fig. 3a–d). Image stacks were combined into a single image was done using Leica Application Suite V4.2. Images were edited using Photoshop CS8 (Version 6.1) (Adobe Inc.). Measurement of holotype and developmental stages of *B. predatorius* were done using AxioVision 4.8. Feeding behaviour of *B. predatorius* was recorded with a hand-held smart phone connected with Leica S8 APO stereozoom trinocular microscope. The rate of feeding of mites by *B. predatorius* larva was estimated from the recordings. The behavioural peculiarities of *B. predatorius* while feeding on the mites was also noted.

### Statistical analysis

Firstly, we analysed whether galls differed in volume between attacked ones and those without *Bracon* larvae (n=109) using Student's t-test with unequal variances. Then we examined the relationship between the number of mites per gall (response variable), numbers of *Bracon* larvae present and gall size measured as diameter × height (explanatory variables). Visual inspection of an initial scatterplot of mite number versus *Bracon* number showed that galls with *Bracon* larvae all had far fewer mites than unattacked galls. Therefore, we performed three separate analyses. Firstly, we compared gall volume for attacked and unattacked galls as well as unattacked galls and those containing a single *Bracon* using two-tailed t-tests with unequal variances. Secondly, we compared mite numbers between unattacked galls and attacked galls as well as against galls containing a single *Bracon* larva. Thirdly, we tested whether in attacked galls there was an effect of numbers of *Bracon* larvae on mite number using ANOVA. To make model errors satisfactorily near normal, we used the transformation log(number of *Bracon* larvae) to reduce overdispersion. Further analyses excluding a few remaining outlier data points made no substantive difference to results. All data analyses were performed using R^37^.

## Supplementary Information

Supplementary Video 1.

Supplementary Legends.

## Data Availability

All data are available in the main text or the supplementary materials.
