# Supplementary material for: Completely predatory development is described in a braconid wasp
Source: Sci Rep. 2022 Feb 2;12:1747. doi: 10.1038/s41598-022-05705-x (PMC8810843; doi:10.1038/s41598-022-05705-x)
Supplement: Supplementary file 2 — Supplementary Legends. [file 41598_2022_5705_MOESM2_ESM.pdf]

**Completely predatory development is described in a braconid wasp**

**A.P. Ranjith[1†], Donald L.J. Quicke[2,3], K. Manjusha[4], Buntika A. Butcher[2,3], & M. Nasser[1*]**

**Supplementary Video 1 (separate file).** Larval predatory behaviour of *Bracon predatorius* Ranjith & Quicke sp. nov.
